# Ex Vivo Immune Response of Casein-Sensitized Lymphocytes to Bovine Milk Protein Composite Genotypes: Beyond the β-Casein A1/A2 Paradigm

**DOI:** 10.3390/nu18183021

**Published:** 2026-09-16

**Authors:** Lidia Hanna Markiewicz, Dagmara Złotkowska, Anna Maria Ogrodowczyk, Ewa Wasilewska, Joanna Fotschki, Kamil Oleński, Stanisław Kamiński, Barbara Wróblewska

**Affiliations:** 1 Immunology and Food Microbiology Group, InLife Institute of Animal Reproduction and Food Research, Polish Academy of Sciences, Trylińskiego 18, 10-683 Olsztyn, Poland; d.zlotkowska@pan.olsztyn.pl (D.Z.); a.ogrodowczyk@pan.olsztyn.pl (A.M.O.); ewa.wasilewska20@gmail.com (E.W.); j.fotschki@pan.olsztyn.pl (J.F.); b.wroblewska@pan.olsztyn.pl (B.W.); 2 Department of Animal Genetics, University of Warmia nad Mazury, Oczapowskiego 5, 10-719 Olsztyn, Poland; kamel@uwm.edu.pl (K.O.); stachel@uwm.edu.pl (S.K.)

**Keywords:** A2A2 milk, milk protein variants, β-casein, κ-casein, β-lactoglobulin, ex vivo, casein sensitization, T lymphocytes, inflammatory response

## Abstract

**Background**: Although the impact of β-casein A1/A2 variants on human health is well-documented, the immunogenic potential of complex milk protein variants, including κ-casein (variants A, B, and E) and β-lactoglobulin (variants A and B) polymorphisms, remains poorly understood. This study aimed to evaluate ex vivo the cellular response of casein-sensitized lymphocytes to digests of milk defined for β-casein, κ-casein and β-lactoglobulin polymorphisms. **Methods**: BALB/c mice were injected intraperitoneally with casein (CAS group) or PBS (control group), and their lymphocytes were subsequently isolated and stimulated with simulated gastrointestinal digests of milk from cows with the genotypes: REF (A1A2/AB/AB), HM1 (A1A1/AA/AA), HM2 (A2A2/BB/BB), HT1 (A1A1/AE/AB), and HT2 (A1A1/BE/AB). We then analyzed the following: T-cell subpopulations (CD4^+^CD25^+^ and CD4^+^Fosp3^+^), proliferation, and the secretion (TNF-α, IL-6, IL-10, IL-17A, IL-4) and gene expression (IL-18, IL-1β, TGFβ1) of key cytokines. **Results**: Lymphocytes from sensitized mice (CAS) showed generally stronger responses to all treatments compared to non-sensitized PBS cells. Of note is the robust pro-inflammatory response of sensitized lymphocytes to HT1 and HT2 digests, as evidenced by a 9- to 10-fold increase in TNF-α and IL-6 secretion compared to the PBS counterparts. This response was uniquely associated with the presence of the κ-casein E variant. Notably, while the population of regulatory lymphocytes (CD4^+^CD25^+^ and CD4^+^Foxp3^+^) expanded significantly in casein-sensitized lymphocytes in response to all hydrolysates, this expansion was markedly impaired in PBS lymphocytes. **Conclusions**: Genetic polymorphism of milk proteins, particularly the presence of the κ-casein E variant, significantly modulates the pro-inflammatory potential of milk digests in sensitized lymphocytes. However, while these findings suggest new strategies for reducing milk immunogenicity, further mechanistic research is required to validate the role of the specific composition of protein variants in this phenomenon.

## 1. Introduction

Cow milk is one of the most important animal-originating components of human nutrition, particularly during infancy, where it serves as a primary source of essential nutrients. Among its constituents, proteins play a pivotal role in supporting the physiological development of an organism. Despite their beneficial attributes, milk proteins are recognized as common dietary allergens, particularly in pediatric populations, affecting 2–3% of infants and young children and about 0.5–3% of adults [1]. Milk proteins consist of about 80% caseins (αs1-, αs2-, β-, and κ-casein) and 20% whey proteins (mainly α-lactalbumin (αLA), bovine serum albumin and β-lactoglobulin (βLG), and allergic reactions have been reported for each of these fractions [2]. To establish the biological relevance of these proteins, it is essential to consider their genetic diversity, which contributes to both the technological properties of milk and its potential immunogenicity.

The milk proteins αs1-casein, β-casein, and κ-casein are encoded by the *CSN1S1*, *CSN2*, and *CSN3* genes, respectively, each with multiple alleles linked to different protein variants. The *CSN1S1* gene has nine alleles (A–I), while β-casein, which makes up to 45% of total casein, has 12 variants, with A1 and A2 being the most common [3]. The *CSN2* B allele is associated with higher casein content and smaller micelle size, enhancing rennet coagulation. κ-casein, encoded by *CSN3*, has at least 14 alleles, with the B variant associated with improved coagulation traits, higher protein content and cheese yield [4]. In contrast to the B allele, the A and particularly the E variants are associated with less favorable milk coagulation properties, including longer rennet coagulation time, weaker curd firmness, and reduced cheese yield. While the A allele is generally considered a neutral or intermediate variant, the E allele additionally affects casein micelle structure and stability, making milk from AE and especially EE genotypes less suitable for cheese manufacture [5,6]. Among whey proteins, β-lactoglobulin (βLG), encoded by *BLG*, has two main variants (A and B), with I, J, and W being rare. α-lactalbumin (αLA), encoded by *LALBA*, occurs mainly in variants A and B, with C being uncommon [3]. This extensive polymorphism suggests that milk from different genotypes may vary not only in technological traits but also in the biological activity of the peptides released during the digestion process.

To date, research on milk protein variants has primarily focused on isolated proteins, particularly the A1 and A2 variants of β-casein. These variants differ at position 67, where a histidine (A1) to proline (A2) substitution significantly alters proteolytic cleavage and the subsequent release of β-casomorphin-7 (BCM-7) [7]. As a bioactive opioid peptide, BCM-7 can delay gastrointestinal transit and promote intestinal inflammation by activating µ-opioid receptors [8]. Clinical studies indicate that A1 milk consumption increases pro-inflammatory markers like myeloperoxidase (MPO) and interleukin-4 (IL-4), often exacerbating symptoms misdiagnosed as lactose intolerance [9,10,11]. In contrast, A2 milk is associated with more favorable metabolic outcomes, higher glutathione concentrations, suggesting a superior redox status [12,13] as well as better gastrointestinal comfort and microbiota [14].

Mechanistically, the histidine-to-proline change determines the accessibility of peptide bonds to proteases and influences casein micelle stability, regulating BCM-7 yield [7,15]. BCM-7 exerts potent immunomodulatory effects; for instance, milk extracts from A1A1 cows upregulate µ-opioid receptor expression while downregulating dipeptidyl peptidase IV (DPPIV)—the primary enzyme for BCM-7 degradation—in PBMCs (peripheral blood mononuclear cells) of children with atopic dermatitis [16]. While these findings underscore how a single amino acid substitution in β-casein alters gastrointestinal physiology and immune modulation, they also highlight a significant knowledge gap. There is a pressing need to explore how broader combinations of milk protein variants, beyond β-casein alone, collectively influence immunogenicity and physiological responses.

Over the last decades, Polish Holstein-Friesian (HF) cattle have undergone significant shifts in milk-protein allele distributions, characterized by a steady decline of the β-casein A1 allele (from 0.40 in 2006 to 0.33 in 2021) and a corresponding rise in the ancestral A2 variant to approximately 67% [17]. High-resolution genotyping of recent cohorts further confirms this trend, alongside a notable enrichment of the κ-casein B allele in bulls (from 19% to 63%) and a subtle but detectable increase in the E variant [17,18]. However, these genetic shifts are complicated by unbalanced protein expression; in A1A2 heterozygotes, the A1 fraction remains nearly seven times more concentrated than A2, underscoring that genotype alone does not fully predict the final proteomic composition of milk [19].

These progressive multi-year trends toward variants associated with improved technological performance and altered nutritional profiles—specifically the A2A2 (*CSN2*) and BB (*CSN3*) genotypes—provided the rationale for our experimental design. While this technologically preferred combination occurs in only a small percentage of the current population, its increasing prevalence necessitates a detailed functional comparison [17]. Consequently, we defined contrasting milk phenotypes for this study, such as HM1 (A1A1/AA/AA) and HM2 (A2A2/BB/BB), alongside HT1 and HT2 types incorporating the κ-casein E variant, to explore the mechanistic differences between these specific protein profiles and their impact on immune recognition.

The aim of the work was to assess ex vivo the relationship between milk protein variants and their immunogenic properties in the mouse model of cow milk protein allergy (CMPA). We hypothesized that genetic polymorphism of main milk proteins, i.e., β-casein, κ-casein and β-lactoglobulin, impacts the cellular response of casein-sensitized immune cells. By integrating multiple protein genotypes rather than focusing on a single variant, this study addresses a critical and understudied aspect of milk allergenicity.

## 2. Materials and Methods

### 2.1. Cows, Genotyping, and Selection of Genotypes

A reference population of 1215 Holstein-Friesian cows, maintained across 25 dairy farms in North-Eastern Poland (Warmia, Mazury, and Podlasie regions), served as the genetic framework for identifying and selecting the specific milk protein genotypes used in this study. Cows were fed according to a standard feeding system based on corn silage and a mix of grain, minerals and vitamins in quantities depending on milk yield. Cows were kept in standard well-being conditions in a barn with free access to the outdoor gated field. This approach ensured uniformity in feeding regime, housing conditions, climate, and herd management, thereby reducing external factors known to influence milk composition and bioactivity [20,21].

Ear tissue samples were collected from the cows using Allflex technology (Allflex Livestock Intelligence Polska, Psary Małe, Polska) and used to isolate genomic DNA using the NucleoSpin Tissue Mini Kit (Macherey-Nagel, Dueren, Germany) according to the manufacturer’s protocols. The ear samples were collected as part of a routine veterinary procedure required for the evaluation of the genomic breeding value of the cows, and therefore, no authorization from the Bioethics Commission was required under Polish law. Cow genotypes were identified using an Illumina Bovine MD Chip (Illumina, San Diego, CA, USA). For the set of SNPs present on the microarray, a preliminary quality data analysis was performed using Illumina GenomeStudio (version 2011.1; Illumina, San Diego, CA, USA) software. SNP clusters that were not separated sufficiently were removed. Subsequently, all SNPs with Call Frequency below 0.99 and clusters with too low a mean normalized intensity signal of genotypes were zeroed. Finally, the correctness of the SNP genotypes was confirmed by checking the concordance between duplicated SNPs on the same chip. In all samples, SNPs representing polymorphic loci at the β-casein, κ-casein and β-lactoglobulin were successfully genotyped.

Based on the frequency of particular gene variants in the available cow herds, variants A1 and A2 of the β-casein gene (*CSN2*), variants A, B and E of the κ-casein gene (*CSN3*), and A, B variants of the β-lactoglobulin gene (*BLG*) were chosen in combinations presented in Table 1. To sum up, five combinations of tested gene variants were selected: a genotype heterozygotic in all loci (REF, which served as the reference milk protein variant set), two homozygotic genotypes, opposite in all three tested loci (HM1 and HM2), and two genotypes that stand out by the presence of the E variant in the *CSN3* locus (AE or BE; HT1 and HT2, respectively). Detailed information on the analyzed polymorphisms, including amino acid substitutions and reference SNP identifiers (rs IDs), is provided in Supplementary Table S1.

### 2.2. Milk Sampling

To further control biological variability, milk was obtained exclusively from clinically healthy cows. Animals were excluded if they exhibited any signs of illness, including apathy, reduced feed intake, moderate to severe lameness, diarrhea, respiratory symptoms (cough and/or nasal discharge), udder swelling or tenderness, abnormal foremilk (e.g., flakes, clots, or blood), visible skin lesions or infections, or if they were undergoing veterinary treatment or had recently received antibiotic therapy. All cows were in their first lactation and in comparable stages of lactation (before the 200th day), as the lactation phase strongly affects protein content, micelle structure, and peptide release during digestion [21,22]. To ensure that the collected milk was representative of each genotype while minimizing the influence of individual animals, milk from three cows was pooled for each genotype group. Before milking, the udder was carefully cleaned to minimize unintended microbial contamination. About 200 mL of fresh milk was collected from the morning milking, proportionally from each parity of the udder, into sterile glass bottles (Simax, Sázava, Czech Republic). Immediately after sampling, the milk was rapidly cooled to 4 °C and transported to the laboratory within 2–4 h. It was then aliquoted and stored at −20 °C for further analyses. Because the sampling involved no intervention, treatment, or manipulation of animals beyond routine milking, the procedure did not require approval from an ethics committee.

### 2.3. Milk Samples Processing

Before simulated digestion, milk samples were defatted to eliminate potential interference from the lipid fraction and ensure that subsequent analyses focused exclusively on the contribution of milk proteins to the biological activity of the resulting digests. The protocol [23] preserved protein composition and integrity. Briefly, milk samples were thawed, pooled in equal proportions, and heated to 40 °C. They were then allowed to equilibrate to room temperature (RT) and centrifuged (15 min, 5000× *g*, RT, fixed-angle rotor). To facilitate phase separation, the samples were cooled to 4 °C. The protein-rich intermediate phase was carefully recovered by aspiration, excluding both the upper lipid layer and the basal pellet. Finally, the protein concentration of the resulting skimmed milk was determined using the Kjeldahl method.

### 2.4. Simulated Gastrointestinal Digestion (SGD) of Milk and Characteristics of the Hydrolysates

A two-step, gastric-intestinal simulated digestion was performed according to the updated INFOGEST 2.0 protocol [24]. The simulated gastric fluid (SGF), simulated intestinal fluid (SIF), and the activity of pepsin and trypsin were prepared as recommended [24]. In the gastric phase of the simulated digestion, to the warmed-up (37 °C) pooled, defatted milk sample (5 mL), 3.75 mL of warm SGF was added, and the pH was adjusted to 3 with 1N HCl. Next, 2.5 µL of 0.3 M CaCl2 and the pepsin solution in SGF buffer (0.8 mL, the final pepsin concentration of 2000 U/mL in the digesting solution) were added, and the volume was set to 10 mL with sterile distilled water. The mixture was incubated for 1 h at 37 °C with gentle mixing. After completing the gastric step, 5.5 mL of warmed simulated intestinal fluid (SIF) was added to the gastric step digest and the pH was set to 7 (1N NaOH). Next, 1.25 mL of bile solution containing 1.25 g of fresh bile extract (B8631, Sigma, Poznań, Poland), 20 µL of 0.3 M CaCl_2_ and 2.5 mL of pancreatin (P-7545, Sigma; the final trypsin concentration of 100 U/mL) were added. The volume was filled up to 20 mL and the whole mixture was incubated at 37 °C for 2 h with gentle mixing. After that time, enzymatic activity was terminated by heating the samples at 100 °C for 5 min. Enzymes and undigested debris were removed by centrifugation at 500× *g*, 4 °C, for 5 min. The resulting supernatant (centrifuged digest, CD) was collected, aliquoted, and stored at −20 °C. Along with milk samples, a sample containing SGF, SIF and enzymes but no protein substrate was processed in parallel and used in further analyses as the enzymatic control of SGD (ENZ).

For the visualization of the digestion process and to confirm the removal of the non-digested proteins, aliquots were taken after the gastric step (GS), the intestinal step (IS), and after the removal of undigested proteins (CD). Next, they were separated by SDS-PAGE (12.05% polyacrylamide with Tris-glycine [25]) in the presence of non-digested pooled milk (ND).

High-performance liquid chromatography (HPLC) with ultraviolet detection (HPLC-UV) was used to determine the peptide profile of the digested samples, following the method of [26]. Samples were diluted (1:2) with 0.1% trifluoroacetic acid (TFA) in deionized water and filtered through a 0.45 μm polyvinylidene difluoride (PVDF) filter. Separation was performed at 1.0 mL/min using a gradient program with phases of (A) 0.1% TFA in water and (B) acetonitrile:water 80:20/0.1% TFA. The gradient started at 100% A, changing linearly to 80% B over 40 min, then equilibrating to 100% A. Analysis was done on a Shimadzu HPLC system (Model LC-8A) with a Jupiter^®^ 5 μm C18 300 Å column, a binary pump, an in-line degasser, a photodiode array, and an absorbance detector set at 214 nm, using LabSolutions software version 5.92. Digested external standards (undefined in terms of genetic variants): β-lactoglobulin (L3908, Sigma), α-casein (C6780, Sigma), κ-casein (C0406, Sigma) and the casein mixture (E414, Sigma) were run for comparison purposes. A schematic overview of the workflow employed for obtaining genotype-defined bovine milk digests is provided in Supplementary Materials (Figure S1).

### 2.5. Mouse Model of Allergy to Casein

BALB/c mice were selected for this study because they represent the established standard model for food-protein sensitization and Th2-mediated allergic responses. Their immunological background is characterized by a robust type-2 polarization, which facilitates IgE class switching, mast-cell activation, and cytokine profiles typical of mucosal food allergy. This strain has been consistently used in experimental models investigating milk-protein allergy, oral sensitization, and antigen-specific lymphocyte activation. Forty female Balb/c mice, purchased at six weeks of age from the Centre of Experimental Medicine in Białystok (Poland), were housed in the animal care facility at the InLife Institute of Animal Reproduction and Food Research, Polish Academy of Sciences in Olsztyn (Poland), and the experiment was conducted according to the published protocols with modifications [27]. For the whole experiment, the mice were fed a milk protein-free maintenance diet (1320 TPF, Altromin, Lage, Germany), and feed and water were provided ad libitum. Animals were housed in individually ventilated cages (IVC system, M.A.C.S.^®^, Alternative Design Manufacturing and Supply Inc., Siloam Springs, AR, USA) with a 15-air-change-per-hour ventilation rate. Five mice were housed per cage under controlled environmental conditions (22–24 °C, 45–64% relative humidity) with a 12 h light/dark cycle. Sterilized, dust-free beech wood shavings were used as bedding. Environmental enrichment consisted of shelters, tunnels, and nesting material. Animals were allocated to two experimental groups (*n* = 20 each) to ensure comparable age and body weight distributions; no formal randomization procedure was applied. The sensitized group (CAS) received intraperitoneal injections of 100 μg casein (E3414, Sigma) emulsified in Freund’s adjuvant (total injection volume: 200 µL), while the non-sensitized control group (PBS) received an equivalent volume of PBS. Three injections were made weekly starting from the experiment’s first day. The fecal and blood samples were collected on the 1st, 14th and last day of the experiment to monitor the specific humoral response. Animals were terminated on the 32nd day via CO_2_ inhalation [27,28]. All procedures applied in the experiment were approved by the Local Ethics Committee in Olsztyn (approval no. 16/2021). Throughout the study, animals were monitored daily for general health status, activity, body condition, grooming behaviour, and signs of discomfort. All procedures were classified as moderate severity according to the approved protocol. To minimize handling-related stress, mice were regularly accustomed to handling throughout the experiment. No unexpected adverse events occurred during the study. No predefined humane endpoints requiring early euthanasia were established, as animals remained in good clinical condition throughout the experimental period and no signs requiring premature termination were observed. No animals were excluded from the experiment, and all animals were included in the final analyses. A schematic overview of the murine sensitization and ex vivo splenocyte-response workflow is provided in Supplementary Materials (Figure S2).

### 2.6. Determination of Antibody Levels in Blood Serum and Feces

The titers of specific IgG and IgA in blood serum and sIgA in feces were determined with the standard indirect ELISA method according to the protocol by Złotkowska et al. [27]. Briefly, 96-well plates were coated with casein (20 µg/mL) and blocked with 1% BSA. After triple washing with PBST (PBS with 0.5% Tween 20), serial dilutions of serum samples or fecal extracts were loaded onto plates and incubated (overnight, 8 °C). Next, the washing procedure was repeated and horseradish peroxidase (HRP)-labeled anti-mouse IgG or anti-mouse IgA (Sigma) was added and incubated for 1 h at 8 °C. The reaction was developed with the use of the substrate ABTS (Millipore, Temecula, CA, USA), and absorbance at λ = 405 nm (Jupiter UVM-340 spectrophotometer, ASSYS Hitech GmbH, Eugendorf, Austria) was read after 1 h incubation. Endpoint titer (EPT) values were expressed as the reciprocal dilution of the last sample dilution of 0.1 OD (optical density) above the negative control [27].

The level of total immunoglobulin class E (IgE) was determined in terminal blood serum samples. The analysis was conducted using a Mouse IgE ELISA Kit (157718, Abcam, Cambridge, UK).

### 2.7. Isolation of Lymphocytes

The procedure applied for the isolation of lymphocytes from mice was previously described in detail [2,27]. In short, spleens, mesenteric lymph nodes (MLN) and Peyer’s patches (PP) were homogenized in a glass douncer in IM medium (RPMI-1640 medium with 1 mM HEPES, 1 mM sodium pyruvate, 10 units/mL penicillin-streptomycin solution), filtered (80 µm nylon filter) and centrifuged (413× *g*, 10 °C, 10 min). Additionally, splenocytes were incubated for 5 min. with the red blood cell lysis buffer (555899, BD Bioscience; Warszawa, Poland). Next, the cells were washed in IM and centrifuged. Peripheral blood mononuclear cells (PBMCs) were isolated using the Leucosep^®^ tubes according to the manufacturer’s protocol (163288, Greiner Bio-On, Kremsmünster, Austria). After the last washing step, cells were re-suspended in 1 mL of IM medium and counted using the Trypan blue method.

For the ex vivo experiment, splenocytes from each experimental mouse group were pooled (7, 7, and 6 each), resulting in three replicates in the CAS and PBS groups. Pooling the splenocytes from CAS and PBS mice standardized the cellular material and allowed the generation of lymphocyte populations representing the sensitized (CAS) and non-sensitized (PBS) immune status. This approach ensured that the observed ex vivo responses reflected group-level immunological characteristics rather than random variation among individual animals.

### 2.8. Lymphocyte Phenotyping

Cells isolated from spleens, MLNs, PPs and PBMCs were subjected to standard protocols applied for cellular and intracellular staining described in detail previously [27,29]. In short, the fluorochromes used included PerCp-Cy5.5 anti-mouse CD4, APC-Cy7 anti-mouse CD8a, PE anti-mouse CD25, AF-488 anti-mouse FOXP3, and APC anti-mouse IL-10; all were purchased from BD Pharmingen (San Diego, CA, USA). Intracellular staining was performed after surface staining using the BD Fixation/Permeabilization Kit (554714, BD Biosciences, Warszawa, Poland) according to the manufacturer’s protocol. The samples were analyzed using a BD LSR Fortessa Cell Analyzer (BD Biosciences, San Jose, CA, USA) equipped with DIVA software version 9.0 (BD Biosciences, San Jose, CA, USA). The data were analyzed using FlowJo 10.3 (FlowJo, LLC, Ashland, OR, USA) software. Lymphocytes were initially gated based on forward scatter (FSC) and side scatter (SSC) characteristics to identify small, low-granularity cells. Doublets were excluded using FSC area versus FSC height parameters. Within the lymphocyte gate, CD4^+^ and CD8^+^ T-cell subsets were identified, and CD4^+^CD25^+^ cells were further gated to determine the Foxp3^+^ regulatory T-cell population. IL-10^+^ cells were quantified within the total CD4^+^ T-cell compartment. The analyzed T-cell subsets were selected to characterize both effector and regulatory arms of the immune response. CD4^+^ T cells represent the major population involved in antigen recognition, whereas CD4^+^CD25^+^ and CD4^+^Foxp3^+^ cells were assessed as markers of regulatory T-cell responses associated with the maintenance of immune tolerance and modulation of allergic inflammation.

### 2.9. Ex Vivo Experiment

Splenocytes from both CAS and PBS mice were plated onto 96-well plates at a concentration of 5 × 10^5^ cells/100 μL in CM medium (IM medium additionally supplemented with 10% heat-inactivated fetal bovine serum and 1 mM of nonessential amino acids). The cultures were supplemented with the digests of milks (HM1, HM2, HT1, HT2, and REF) and an enzymatic control (ENZ) at a concentration of 100 µg/mL. Cells stimulated with concanavalin A (ConA, 10 µg/mL; 234567M, Sigma) or treated only with medium served as controls. After incubation (48 h, 37 °C, 5% CO_2_), cells and cell supernatants were collected for gene expression and cytometric analyses.

### 2.10. Splenocyte/Lymphocyte Proliferation

Analysis of lymphocyte proliferation after the ex vivo experiment was carried out in 96-well plates with the use of the cell proliferation reagent Wst-1 reagent (Roche) as described previously [25]. Briefly, after the experiment was completed, 10 µL of the reagent was added to each well, and the absorbance was read at 450 nm. Results were expressed as the proliferation rate (PR), calculated as follows: ((absorbance of the sample)/(absorbance of the control) × 100%). Cells treated with concanavalin A served as a positive control of cell stimulation. Lymphocyte proliferation was used as an indicator of antigen-specific cellular activation and responses. Enhanced proliferative activity following stimulation reflects the ability of previously sensitized immune cells to recognize and respond to antigenic peptides.

### 2.11. Secretion of Cytokines by Cultured Lymphocytes

The analysis of cytokines secreted by the lymphocytes after 48 h incubation with milk digests was carried out with the use of the BD Cytometric Bead Array Mouse Th1/Th2/Th17 Cytokine Kit (560485, BD Biosciences) that enabled the determination of IL-2, IL-4, IL-6, IL-10, interferon-gamma (IFN-γ), tumor necrosis factor (TNF-α), and IL-17A. The obtained results were analyzed using FCAP Array 3.0 software (BD Biosciences) [27]. The selected cytokines were analyzed to assess different aspects of immune activation. TNF-α and IL-6 were included as markers of pro-inflammatory responses, IL-17A as an indicator of Th17-associated activity, whereas IL-2, IL-4, IL-10, and IFN-γ were measured to evaluate T-cell activation, type 2 immune responses, regulatory mechanisms, and Th1-related responses, respectively.

### 2.12. RNA Isolation and Gene Expression

After completion of the ex vivo experiment, total RNA was isolated from CAS lymphocytes using the Universal RNA Purification Kit (Eurx, Gdańsk, Poland), and 100 ng of RNA was reverse transcribed with the smART First Strand cDNA Synthesis kit (Eurx). Real-time PCR reactions were carried out in a QuantStudio 6 Flex system (Thermo Fisher Scientific, Life Technologies, Warszawa, Poland) using SYBR Green chemistry (PowerUp™ SYBR™ Green Master Mix, Thermo Fisher Scientific). Amplifications were performed as described previously [30] using gene-specific primer pairs (Table S2). Changes in the expression of tested genes were calculated using the ΔΔCT method and *ACTB* as a reference gene in the Thermo Fisher Scientific Cloud using a Relative Quantity app. Expression of *Il18*, *Il1b* and *Tgfb1* was evaluated to complement cytokine secretion analyses and to gain additional insight into pathways involved in inflammatory activation (IL-18, IL-1β) and immune regulation or tolerance induction (TGFβ1), which were not covered by the cytokine panel used in this study.

### 2.13. Statistical Analysis

Statistica software version 14.1 (StatSoft, Kraków, Poland) was used for statistical analyses. The Shapiro–Wilk test and the Brown–Forsythe test were used to verify the normality and homoscedasticity of the variables, respectively. Two-way ANOVA was performed to evaluate the effect of the applied Treatment (milk digests: REF, HM1, HM2, HT1, HT2, and ENZ) and the sensitization state of lymphocytes (Group; CAS and PBS) on their response, as well as the Treatment × Group interaction. A post-hoc Tukey test or a non-parametric Kruskal–Wallis test (for data lacking normality or homoscedasticity) was applied.

## 3. Results

### 3.1. Selection of Casein and β-Lactoglobulin Variants

The investigated milk protein variants were selected based on the frequency of detected genotypes in tested herds and the availability of milk samples (summer 2021). It should be stressed that by standardizing farm origin, seasonality, breed, health status, lactation stage, milking method, and hygienic procedures, and by sampling multiple animals per genotype, the study design minimized external sources of variation. This ensured that observed differences in the immunogenic properties of milk digests could be attributed primarily to milk protein polymorphisms, which were the central focus of the investigation.

### 3.2. Simulated Gastrointestinal Digestion and Characteristics of Digests

Simulated gastrointestinal digestion (SGD) was performed on defatted milk to eliminate the potential interference of the lipid fraction on the tested immune parameters [31]. Protein concentration of the defatted milk pools ranged from 3.04% to 3.05% in HT1 and HM1, to 3.22% and 3.23% in HT2 and HM2, and to 3.40% in REF milk. The procedure resulted in the hydrolysis of high-molecular-mass proteins across all tested samples (Figure S3). The profiles of the soluble digests obtained are shown in the chromatograms (Figure S4).

### 3.3. Mouse Model of Sensitization to Caseins

**Figure 1:**
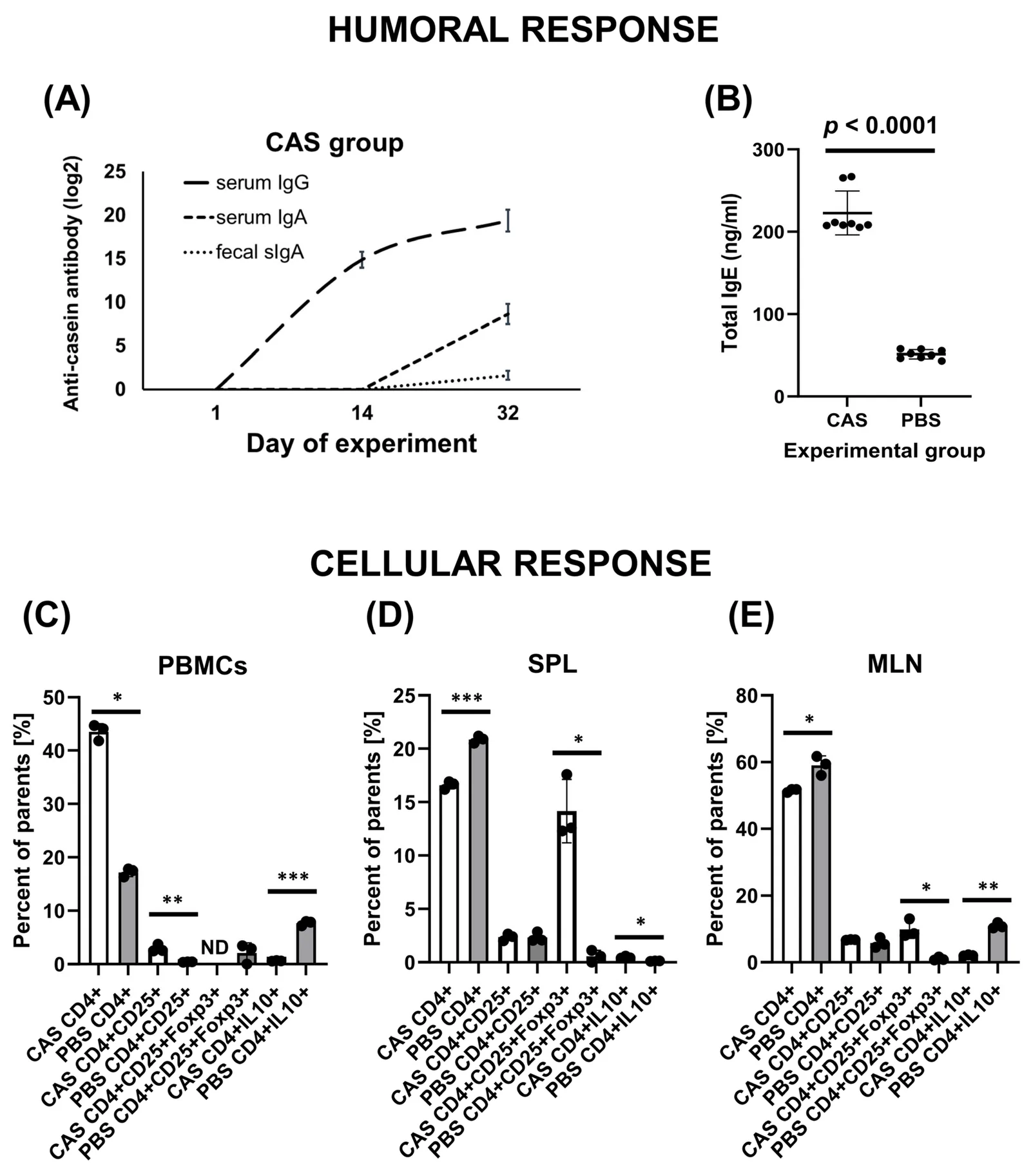
Humoral and cellular response to casein in a mouse model of sensitization to casein. Humoral response of mice: (**A**) development of specific serum IgG and IgA and fecal sIgA in mice sensitized to casein (CAS group); and (**B**) total serum IgE level in mice sensitized to casein (CAS) and in PBS-treated mice (PBS) after 32 days post-immunization. Cellular response of CD4^+^ cells: (**C**) peripheral blood mononuclear cells (PBMCs), (**D**) lymphocytes isolated from spleen (SPL), and (**E**) mesenteric lymph nodes (MLN). ND—not detected. *, ** and *** denote statistically significant differences at *p* < 0.05, 0.01 and 0.001, respectively.

Analysis of the humoral response showed elevated anti-casein IgGs (Figure 1A) and increased total IgE levels in casein-immunized mice compared to the PBS group (222.87 ± 26.7 vs. 51 ± 6 ng/mL, respectively; *p* < 0.0001, Figure 1B), confirming successful allergic sensitization. This humoral response was supported by the cellular findings—in the inductive tissue (MLN), the immunization markedly increased the percentage of CD4^+^CD25^+^Foxp3^+^ regulatory T cells (about 3-fold), and in the effector tissue (SPL), about 10-fold higher elevation was observed compared to the PBS group (Figure 1D,E). These results suggest that CAS treatment enhances the regulatory T cell population, potentially modulating allergic responses in casein-immunized mice.

The PBMCs reflect the systemic circulation of effector cells. In the presented experiment, PBMCs from CAS-treated mice displayed features consistent with sensitization, including a predominance of CD4^+^ T cells and an increased percentage of CD4^+^CD25^+^ regulatory cells. These cells lacked Foxp3 expression and did not produce detectable IL-10 (Figure 1C). Losing IL-10 may contribute to the exacerbation of allergic responses, highlighting the importance of regulatory T cells in maintaining immune tolerance during sensitization.

### 3.4. Ex Vivo Stimulation of Splenocytes with Milk Digests

**Figure 2:**
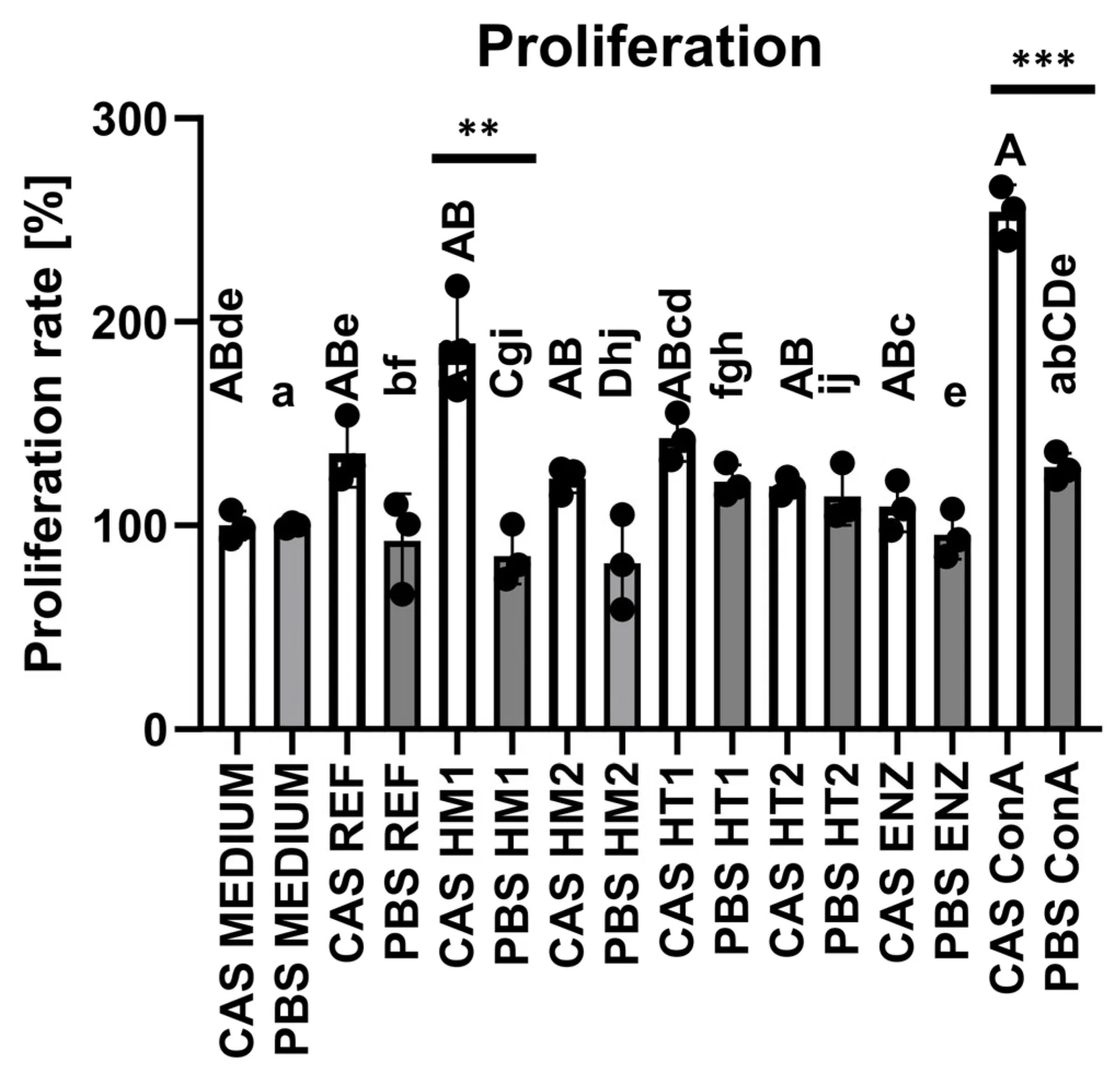
Proliferation rate of cultured splenocytes isolated from casein-sensitized (CAS) and non-sensitized (PBS) mice. Splenocytes were treated with digests of the tested variants of cow milk (HM1, HM2, HT1, HT2, and REF), concanavalin A (ConA, positive control) and ENZ (enzymatic control of digestion) or with medium. Proliferation was calculated as the percentage of absorbance readings for cells cultured in medium and is presented as the mean ± CV%. Differences between CAS and PBS lymphocytes treated with the same factor marked with ** and *** indicate statistical significance (*t*-test) at *p* < 0.01 and 0.001, respectively. Differences between lymphocytes isolated from the same group (CAS or PBS) treated with different factors (REF, HM1, HM2, HT1, HT2, ENZ, ConA) are marked with the same small letter for values significantly different at *p* < 0.05 or with the same capital letter for *p* < 0.001.

The basic parameter describing the response of lymphocytes to stimulation is their ability to proliferate upon exposure to specific or nonspecific factors. In our study, the proliferation rate (PR) of CAS and PBS lymphocytes cultured in medium only was comparable. Still, a trend for elevated proliferation in CAS lymphocytes was observed regardless of the treatment applied (Figure 2). A significantly elevated PR was observed only for HM1, which markedly increased proliferation in sensitized cells (190% for CAS vs. 85% for PBS cells, *p* < 0.01), similarly to the effect observed in non-specific mitogen concanavalin A-treated cells (254% vs. 129%, *p* < 0.001) (Figure 2).

Statistical analysis of the effect of the tested digests within one lymphocyte group showed that PR of sensitized (CAS) cells treated with HM2, HT2 and ENZ was comparable to that of splenocytes cultured in medium alone and to cells treated with the reference milk digest (REF; Figure 2). In contrast, ConA and HM1 induced the highest PR increase (254% and 190%, respectively), significantly exceeding all other treatments and controls. Digests of the reference milk (REF) and HT1 significantly increased PR when compared to non-treated cells (Figure 2), whereas HT1 also had the same effect in comparison to HT2- and ENZ-treated splenocytes.

Unlike the CAS splenocytes, the non-sensitized splenocytes (PBS) exhibited a weaker response to the applied treatments (Figure 2), and statistically higher PR (129%; *p* < 0.05) was noted only for splenocytes treated with HT1 (compared to REF, HM1, HM2 and HT2) and for ConA (compared to all other treatments except for HT1 and HT2).

### 3.5. Ex Vivo Response of Lymphocytes to Digested Milks

**Figure 3:**
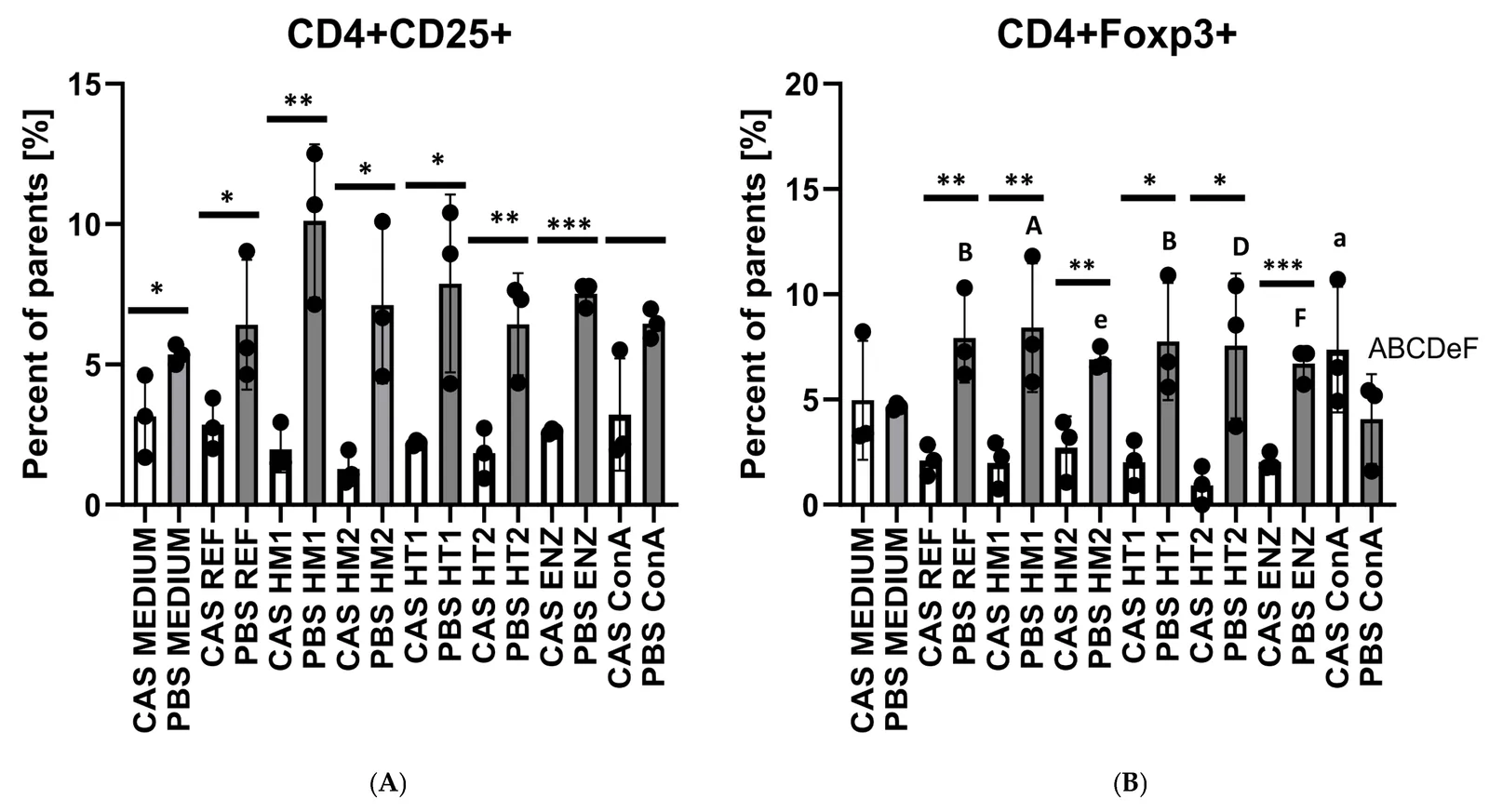
Ex vivo stimulation of casein-sensitized (CAS) and non-sensitized (PBS) splenocytes with digests of the tested cow’s milk variants. (**A**,**B**)—percentages of CD4^+^CD25^+^ and CD4^+^Foxp3^+^ T cells following treatment with milk digests (REF, HM1, HM2, HT1, HT2), medium alone, enzymatic control (ENZ), or concanavalin A (ConA). Differences between CAS and PBS lymphocytes treated with the same factor marked with *, ** and *** indicate statistical significance (*t*-test) at *p* < 0.05, 0.01 and 0.001, respectively. Differences between treatments within the same experimental group are marked with the same small letter for values significantly different at *p* < 0.05 or with the same capital letter for *p* < 0.01.

The performed phenotypic analysis confirmed a markedly stronger activation of splenocytes from casein-sensitized mice (CAS), reflected by a consistently higher proportion of CD4^+^CD25^+^ T cells across all treatments compared with PBS lymphocytes (Figure 3A). Similarly, the frequency of regulatory CD4^+^Foxp3^+^ T cells was significantly elevated in CAS splenocytes relative to the PBS group, although this effect was evident primarily upon stimulation with milk hydrolysates (Figure 3B).

When the effects of the hydrolysates were compared within each lymphocyte group, sensitized splenocytes displayed a uniformly stronger response to ex vivo stimulation. Although differences among treatments in CAS cells did not reach statistical significance, all hydrolysates induced a clear upward trend in the CD4^+^CD25^+^ population (Figure 3A). A similar trend was observed for the CD4^+^Foxp3^+^ subset, which increased following stimulation with each hydrolysed milk variant, reaching statistical significance only in response to ConA.

In contrast, splenocytes from non-sensitized PBS mice were characterized by uniformly low (<5%) frequencies of CD4^+^CD25^+^ T cells, irrespective of treatment (Figure 3B). The CD4^+^Foxp3^+^ population in PBS splenocytes was highest in the ConA-treated samples, while stimulation with any of the milk hydrolysates resulted in consistently low values not exceeding 3%.

**Figure 4:**
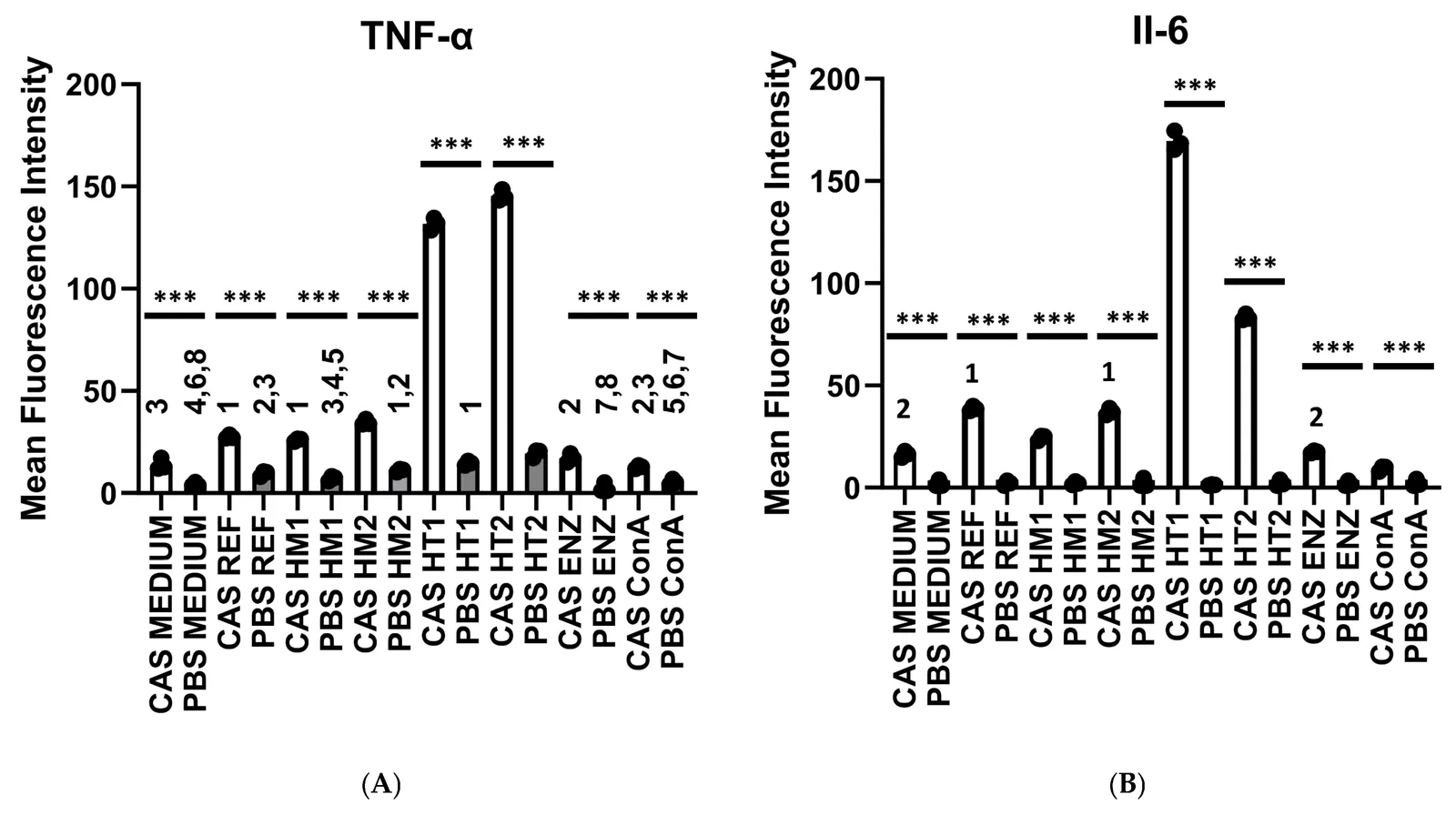
Secretion of cytokines by casein-sensitized (CAS) and non-sensitized (PBS) lymphocytes stimulated ex vivo with digests of the tested cow’s milk variants. Mean fluorescence intensity values recorded for TNFα (**A**) and IL-6 (**B**) produced by lymphocytes following treatment with milk hydrolysates (REF, HM1, HM2, HT1, HT2), medium alone, enzymatic control (ENZ), or concanavalin A (ConA). Significant differences between corresponding treatments in CAS and PBS lymphocytes are indicated with *** (*p* < 0.001). Intra-group statistical analysis for TNFα (**A**) in both CAS and PBS lymphocytes, and for IL-6 (**B**) in CAS lymphocytes, revealed significant differences (*p* < 0.01) for the majority of comparisons; for clarity, non-significant differences are marked with identical digits. In contrast, intra-group analyses for IL-6 secretion in PBS lymphocytes did not show statistically significant differences.

When cytokine profiles were analyzed in the post-culture medium, concentrations of most analytes, particularly IL-17A and TNFα, were frequently below the assay’s limit of quantification despite being detectable. Therefore, the results are presented as mean fluorescence intensity (MFI) rather than calculated concentrations to avoid reporting values with a high degree of uncertainty. IL-10 and IFN-γ levels were below detection limits and are not shown. The basal secretion of pro-inflammatory TNF-α and IL-6 in non-treated cells (CAS medium vs. PBS medium, Figure 4A,B) was significantly higher in CAS lymphocytes (*p* < 0.001). This pattern persisted across all treatments, with the strongest responses observed in HT1- and HT2-treated CAS lymphocytes, where TNFα and IL-6 levels exceeded those in control medium by 9- to 10-fold (TNF-α) and 9- to 5-fold (IL-6), respectively. Such a release of the pro-inflammatory TNF-α and IL-6 exclusively in lymphocytes from sensitized mice is a clear indicator of the recall response of T cells, specifically orchestrated through the NF-κB signaling pathway upon re-exposure to milk protein epitopes [32]. This robust cytokine signature confirms that the HT1 and HT2 digests retain immunogenic epitopes capable of inducing a vigorous effector response, mediated by the NF-κB signaling pathway, which acts as the master regulator of pro-inflammatory genes, including TNFα and IL-6, in activated T cells [33].

In our study, IL-17A levels did not differ significantly between PBS and CAS lymphocytes (Figure S5), although a tendency toward elevated IL-17A was observed in sensitized lymphocytes treated with HM1, HM2, HT1, HT2 digests and medium alone. Nevertheless, the disproportionate increase in TNF-α and IL-6 secretion, combined with the absence of a significant increase in cytokines associated with type 2 immune responses (IL-4 and IL-2; Figure S5), suggests that these milk variants primarily trigger acute inflammatory signaling rather than fully activating the canonical allergic cascade. This points toward a distinct, variant-specific mode of immune activation.

### 3.6. Gene Expression in Activated Lymphocytes

Expression of genes coding IL-18 (*Il18*), IL-1β (*Il1b*), and TGFβ1 (*Tgfb1*) was determined in CAS lymphocytes, as these factors were not included in the applied cytokine test (Figure S6). Among the tested genes, only the expression of *Il1b* was elevated (*p* < 0.05) in HT2-treated CAS lymphocytes compared to REF and HM2, confirming a possible mechanism of pro-inflammatory activation of lymphocytes through the NF-κB signaling pathway [33].

### 3.7. Statistical Analysis of Variance Components

To quantify the relative contributions of lymphocyte sensitization state (group) and type of hydrolyzed milk (treatment) to the overall variability of cellular phenotypes and cytokine responses, a variance decomposition analysis (η^2^) was performed (Table 2). The analysis revealed that treatment was the predominant determinant of several key functional readouts, accounting for a substantial proportion of the total variance in proliferation (η^2^ = 0.30, *p* < 0.0001), TNF-α secretion (η^2^ = 0.45, *p* < 0.0001), and IL-6 production (η^2^ = 0.34, *p* < 0.0001). These results indicate that the properties of the milk variant hydrolysates exerted a strong modulatory effect on the pro-inflammatory activity of sensitized lymphocytes.

In contrast, the sensitization state of lymphocytes exhibited its strongest influence on regulatory T-cell subsets. Notably, CD4^+^CD25^+^ (η^2^ = 0.69, *p* < 0.0001) and CD4^+^Foxp3^+^ populations (η^2^ = 0.40, *p* < 0.0001) were highly dependent on prior sensitization, highlighting the central role of immunological background in shaping the regulatory phenotype. Moderate contributions of the group were also observed for IFN-γ (η^2^ = 0.06, *p* = 0.0403). Conversely, several cytokines (IL-4, IL-2, and IL-10) displayed negligible η^2^ values and non-significant *p*-values for both main effects, indicating that these mediators remained largely unaffected under the examined conditions, with their variability dominated by residual (unexplained) variance.

A robust interaction effect (group × treatment) was identified for proliferation (η^2^ = 0.20, *p* < 0.0001), CD4^+^Foxp3^+^ cells (η^2^ = 0.34, *p* < 0.0001), IL-6 (η^2^ = 0.35, *p* < 0.0001), and TNF-α (η^2^ = 0.30, *p* < 0.0001). This indicates that the cellular response to distinct milk hydrolysates was non-additively shaped by the lymphocyte sensitization state, demonstrating that treatment effects were markedly context-dependent. Together, these results underscore the importance of both intrinsic immunological history and extrinsic stimuli in determining the magnitude and direction of lymphocyte activation signatures.

## 4. Discussion

### 4.1. Milks and Digests

The primary objective of this study was to evaluate the immunogenic potential of milk protein composite genotypes, moving beyond the traditional A1/A2 β-casein paradigm. We selected contrasting milk phenotypes, specifically HM1 (A1A1/AA/AA) and HM2 (A2A2/BB/BB), along with HT1 (A1A1/AE/AB) and HT2 (A1A1/BE/AB) that include the κ-casein E variant, to investigate the differences between these protein profiles and their effects on immune recognition. Notably, analysis of our in-house genotyping database comprising over 1500 cows revealed that animals carrying the κ-casein EE genotype consistently also exhibited the A1A1 β-casein genotype, indicating a strong genetic linkage between these variants in the studied population (unpublished data). However, analyses of Holstein populations exceeding one million bulls and cows have revealed the presence of additional β-/κ-casein haplotypes, with A1E and A2E occurring at frequencies of 9.3% and 1.4%, respectively, suggesting that such associations can be detected in larger populations [34]. Although the milk protein variant compositions were carefully chosen to represent meaningful biological differences, they were selected from the available lactating cows and therefore do not cover the full range of polymorphisms found in dairy cattle populations. Nevertheless, to ensure that the observed immunological effects were strictly attributable to genetic polymorphisms, the experimental design strictly controlled for environmental and physiological factors. By standardizing farm origin, breed, seasonality, and lactation stage, we minimized external variables that typically influence milk composition and bioactivity.

In this study, before digestion, milks were defatted to eliminate the potential interference of lipids on immune parameters, ensuring that the biological activity of the resulting digests was derived solely from the protein fraction. It should be noted, however, that despite the milk skimming procedure, a small portion of milk lipids remains dispersed in the aqueous phase. These consist mainly of polar milk fat globule membrane (MFGM) derived lipids such as phosphatidylcholine, phosphatidylethanolamine, phosphatidylserine, phosphatidylinositol, and sphingomyelin, which are known to partition partly into skim milk and whey rather than being fully confined to intact fat globules [35]. In addition, trace amounts of mono- and diacylglycerols, free fatty acids, and sterols persist in the serum phase due to their partial distribution outside the fat globule core or the release of membrane fragments during processing [36]. However, it should be emphasized that the digestion of skim milk does not fully reflect the physiological digestion of whole milk. In native milk, proteins coexist with milk fat globules and MFGM components, which may affect enzyme accessibility, matrix structure, and interactions among milk constituents during gastrointestinal digestion [37,38]. Furthermore, evidence suggests that milk lipids directly influence protein hydrolysis kinetics and peptide release [39]. Consequently, the present model represents a simplified approach focused on the protein fraction, and future studies should investigate how the native lipid matrix interacts with milk protein polymorphisms to shape immune responses.

We employed the standardized INFOGEST simulated gastrointestinal digestion protocol [24] and subsequent processing of the digests that ensured the generation of soluble fractions, the biological activity of which was elucidated through their ability to trigger cellular responses. This approach approximates the physiological conditions in which food proteins undergo gastrointestinal digestion before interacting with host immune cells in their hydrolyzed form. Peptide profiles of digested milk proteins have been described in the literature [40,41,42,43,44]. Our aim was therefore not to re-establish peptide composition, but to assess how the obtained digests generated from naturally occurring milk protein variant composites influence immune activation in the context of casein sensitization. Of particular relevance in this context is the work of Lisson et al. [43,44], who demonstrated in silico and in vitro that susceptibility to gastrointestinal digestion is significantly modulated by amino acid sequence polymorphisms across α-, β-, and κ-casein variants [43,44]. Such structural variations alter the accessibility of epitopes capable of binding to immunoglobulin E (IgE), thereby mediating the initiation of the allergic response. Specifically, digestion of β-casein A1, A2, and B variants generated distinct peptide fragments overlapping with known IgE-binding epitopes. For example, peptides spanning residues 108–129 in β-casein A1 and A2 and residues 103–123 in β-casein B corresponded to the IgE-binding epitope located within residues 107–120. Similarly, peptides encompassing residues 59–72, 59–80, and 58–80 overlapped with another known IgE-binding region (residues 55–70) [43]. In κ-casein, variant-specific peptides spanning residues 136–149 (variants A and E) and 134–150 (variant B) corresponded to the IgE-binding epitope located within residues 137–148 [43]. Comparable observations were reported for αs1- and αs2-casein variants, where digestion-generated peptides spanning residues 174–193 (αs1-casein B), 179–198 (αs1-casein C), 7–29 (αs2-casein A), and 1–22 (αs2-casein B) overlapped with previously identified IgE-binding epitopes [44]. Although variant-specific digestion studies are considerably less abundant for β-lactoglobulin than for caseins, structural differences between A and B variants are known to affect protein conformation and antigenicity [45]. Furthermore, several studies have demonstrated that gastrointestinal digestion of β-lactoglobulin releases peptides corresponding to known IgE-binding regions, including residues 43–68 and 122–146, highlighting the potential contribution of β-lactoglobulin-derived peptides to the overall immunological properties of digested milk [46,47]. Together, these findings demonstrate that even small sequence differences can substantially alter the repertoire of digestion-derived peptides and the presentation of allergenic epitopes, supporting the concept that naturally occurring combinations of milk protein variants may generate biologically distinct peptide mixtures with different immunological properties.

However, most studies conducted to date have focused on isolated proteins or single genetic polymorphisms, providing valuable mechanistic insight but only partially reflecting the biological complexity of native milk, in which multiple protein variants coexist and undergo digestion simultaneously. While the literature provides a comprehensive description of the technological characteristics and certain biological activities of individual milk protein variants [45,48], studies evaluating naturally occurring combinations of β-casein, κ-casein, and β-lactoglobulin variants remain scarce. Consequently, little is known about how interactions among peptides released from different protein fractions collectively influence the immunogenic properties of digested milk. To our knowledge, no previous study has investigated the immune response to milk characterized by defined composite genotypes of β-casein, κ-casein, and β-lactoglobulin under conditions mimicking gastrointestinal digestion.

### 4.2. Immune Response

The induction of an IgE-mediated allergic response is a complex process involving multiple cells and mediators. It comprises two stages: primary sensitization, triggered by initial allergen exposure, and the effector phase, marked by allergic symptoms upon re-exposure [49]. During sensitization, antigen-presenting cells—mainly dendritic cells—internalize milk protein peptides and present them to naïve CD4^+^ T cells, which differentiate into T-helper (Th0) cells. In predisposed mice, Th0 differentiate into Th2 cells, which secrete cytokines such as IL-4 and IL-13, promoting B-cell class switching and the production of allergen-specific IgE [50]. Upon subsequent exposure, IgE bound to FcεRI receptors on basophils and mast cells recognizes allergenic epitopes. This cross-linking triggers the release of mediators, including histamine, causing immediate hypersensitivity reactions [49]. In contrast to Th2 cells, regulatory T cells (Tregs) play a critical role in establishing and maintaining tolerance to dietary antigens. Through the secretion of immunosuppressive cytokines, including IL-10 and TGFβ, Tregs suppress Th2 responses, limit IgE production, and promote immune tolerance. Impaired Treg number or function has been associated with the development and persistence of food allergy [51,52].

Our mouse model confirmed the development of casein sensitization, as demonstrated by both humoral and cellular response markers (Figure 1). Lymphocytes isolated from casein-sensitized mice (CAS) and control mice (PBS) were utilized to evaluate ex vivo the immune response to various bovine milk protein variant combinations. Under ex vivo conditions, when lymphocytes encounter an antigen or epitope that has previously induced sensitization in vivo, they undergo rapid clonal expansion, resulting in distinct and measurable shifts in T-cell subset composition that reflect the activation of specific effector or regulatory subsets [53].

In our study, following exposure to digested milk, CAS lymphocytes displayed a pronounced overrepresentation of regulatory T cells (CD4^+^CD25^+^ and CD4^+^Foxp3^+^) compared with PBS counterparts. Such differences likely reflect the presence of antigen-specific immunological memory in casein-sensitized mice, which, upon re-exposure to casein-containing digests, triggers a compensatory expansion of regulatory T-cell subsets [54]. Moreover, chronic allergic sensitization is known to drive adaptive increases in Foxp3^+^ Tregs as part of a homeostatic feedback loop aimed at restraining excessive type 2 immune responses [55]. In contrast, spleen cells from PBS controls lack antigen-experienced Treg populations and therefore exhibited only baseline regulatory frequencies despite identical culture conditions.

In the present study, the cytokine profile induced by milk digests in CAS splenocytes indicates a distinct, non-canonical pattern of immune activation. While we observed a significant secretion of pro-inflammatory markers such as TNF-α and IL-6, this response was notably dissociated from the type 2 immune response typically associated with allergic reactions, as indicated by the lack of IL-4 and IL-13 secretion. TNF-α and IL-6 are recognized as central orchestrators of early-stage inflammatory processes [56,57]; however, their elevation in the absence of IL-2 suggests that the stimulation by the digests was insufficient to drive full T-cell clonal expansion. This further implies that, although enzymatic hydrolysis spared certain motifs that activate innate-like immune components, it disrupted the specific antigenic epitopes necessary to sustain a mature adaptive allergic response. This cytokine profile highlights the reduced allergenic potential of the tested milk digests, which initiate an inflammatory signal that fails to translate into a productive allergic reaction. It should be noted that the enhanced responses observed in sensitized lymphocytes are not necessarily indicative of direct antigen recognition of all protein variants present in the tested milk digests. Rather, genetic polymorphisms of milk proteins may modify the peptide repertoire generated during digestion, thereby modulating an already primed inflammatory environment through both antigen-specific and non-antigen-specific mechanisms. Therefore, the effects observed for individual milk genotype composites could be interpreted as the net outcome of the biological activity of the entire digestion-derived peptide mixture. Our findings are consistent with previous studies on single milk proteins, whole milk, and milk-derived products, where both the enzymatic hydrolysis and simulated gastrointestinal digestion markedly reduced immunoreactivity and IgE-reactivity towards specific milk proteins [58,59]. Additionally, lactic acid fermentation has been shown to further diminish the immunoreactivity of milk proteins [60].

Although IL-6 and TNF-α are not classical markers of allergic response, their elevated levels are observed in children allergic to cow’s milk proteins even after 6 months of a dairy-free diet and disappearance of allergy symptoms [61]. What is important, this observation was made for both types of allergic mechanisms (IgE-dependent or IgE-independent), which indicates a persistent low-grade inflammatory state due to increased reactivity of the immune system even in the absence of allergenic triggers.

In contrast to our findings, the most frequently described in vitro effect of milk protein-derived peptides is their beneficial effect (including anti-inflammatory and immunomodulatory) [62]. To the best of our knowledge, there are no reports on how hydrolysed allergenic protein complexes affect sensitized immune cells, and only a few studies indicate pro-inflammatory properties of milk hydrolysates or protein fractions, but most often they use simple in vitro cell models of human or murine macrophages (THP-1 and RAW264.7 cells). Chun et al. [63], in their studies on non-digested whey protein concentrates, demonstrated that RAW264.7 macrophages exposed to glycated, rather than native, whey proteins responded with increased expression of pro-inflammatory IL-6, TNF-α, and IL-1β at the level of the LPS-induced response. In studies on human milk, Liang et al. [64] identified specific fractions of human milk digests that enhanced IL-8 pro-inflammatory response of LPS-challenged THP-1-derived macrophages. In the most recent report, low-molecular-weight fractions (smaller than 3 kDa) of digested sweet whey from several species were investigated on LPS-challenged and non-challenged THP-1-derived macrophages [65]. The bovine sweet whey digests increased mRNA expression of proinflammatory CXCL8 (IL-8) in non-challenged macrophages. Importantly, in LPS-challenged macrophages, bovine sweet whey digests additionally induced factors involved in the NF-κB inflammatory pathway (NFKB1, RELA, IL-1β, and CXCL8).

Among milk variants tested in our study, two specifically containing the κ-casein E variant—HT1 (A1A1/AE/AB) and HT2 (A1A1/BE/AB) induced a significantly higher release of both TNF-α and IL-6. Notably, neither HM1 (A1A1/AA/AA) nor HM2 (A2A2/BB/BB) induced a pro-inflammatory response comparable to that observed for HT1 and HT2. Since the strongest cytokine responses were restricted to the two milk digests containing the κ-casein E variant and sharing a comparable AB β-lactoglobulin phenotype, the present data support the involvement of κ-casein E in the observed effect. However, because the investigated milk phenotypes differed in co-occurring protein variants, contributions from the unique peptide composition associated with specific combinations of milk protein variants cannot be completely excluded. Our findings underscore the capacity of milk digests to modulate innate inflammatory signaling, a phenomenon that appears to be highly dependent on the specific peptide profile generated during proteolysis. Recent reports on the technological properties [66], natural modification, and susceptibility to digestion of the variant E of κ-casein highlight its distinct behavior within the milk matrix [67,68]. Specifically, the κ-casein E variant has been associated with altered micellar structures and impaired coagulation properties, which may fundamentally change the accessibility of epitopes during proteolysis [66]. Furthermore, the unique glycosylation and phosphorylation patterns characteristic of this variant may influence its breakdown into bioactive peptides, as recent digestion models suggest that specific κ-casein polymorphisms significantly dictate the release of immunomodulatory sequences [67]. Such structural variations, combined with the presence of other protein variants, likely contribute to the specific inflammatory profile observed in our study.

### 4.3. Limitation of the Study

This study has several limitations that should be considered when interpreting the findings. First, the composite nature of the investigated milk genotypes limits the ability to fully disentangle the contribution of individual protein variants from the cumulative biological activity of the peptide mixtures generated during digestion. Although the strongest ex vivo pro-inflammatory responses were associated with milk digests containing the κ-casein E variant, the possibility cannot be excluded that the observed effect resulted, at least in part, from cumulative activity of peptides derived from β-casein, κ-casein, and β-lactoglobulin.

Another limitation is that the casein preparation used for sensitization was not genetically characterized. At the time the study was conducted, genetically defined casein preparations were not commercially available. Consequently, sensitization was performed using a standard commercial casein preparation, which most likely represented a mixture of the most prevalent bovine casein variants. While this approach was considered appropriate for establishing a broadly casein-reactive immune background, future studies employing genetically defined casein variants may provide additional insight into variant-specific mechanisms of immune recognition.

The use of BALB/c mice constitutes another limitation. This strain is predisposed towards type 2 immune responses and is therefore commonly used in models of IgE-mediated food allergy [69]. Although its selection was intentional and aligned with the objectives of the study, the genetic background of laboratory animals may influence both the magnitude and quality of immune responses. Therefore, validation of the findings in mouse strains with distinct immunological profiles would help establish the broader applicability of the observed effects.

Potential epigenetic mechanisms influencing immune responsiveness were also beyond the scope of the present work. Increasing evidence indicates that epigenetic regulation contributes to the differentiation and function of CD4+ T-cell populations involved in food allergy and immune tolerance [70,71]. Furthermore, splenocytes were pooled within experimental groups to minimize inter-individual variability and obtain sufficient material for the analyses performed. While this approach enabled characterization of group-level responses, it precluded assessment of individual variation, including variability potentially associated with differences in epigenetic programming.

Finally, the study relied on an ex vivo model that provided a controlled platform for investigating immune responses to milk digests differing in protein variant composition. Nevertheless, ex vivo systems cannot fully reproduce the complexity of in vivo immune regulation, including interactions with epithelial barriers, stromal cells, neuroendocrine signals, and microbiota-derived metabolites. In addition, the response to the mitogenic positive control (ConA) was lower than typically reported for freshly isolated murine splenocytes with respect to cytokine secretion, although the strong proliferative response confirmed the viability and responsiveness of the cultured cells. Future studies integrating complementary animal models and in vivo validation will be required to fully elucidate the immunogenic consequences of milk protein diversity.

An additional limitation is that no a priori statistical power calculation was performed. The number of animals was based on previous experiments using the same BALB/c casein-sensitization model and on the amount of biological material required for the planned ex vivo analyses. Although this approach was sufficient to obtain reproducible immune readouts, future studies would benefit from formal power calculations performed during the study-design phase.

## 5. Conclusions

The study demonstrates that the immunogenicity of cow milk extends beyond the β-casein A1/A2 paradigm, with other protein polymorphisms playing a crucial role in modulating the immune response. The κ-casein E variant (present in HT1 and HT2 milk) appears to be a potent pro-inflammatory factor, inducing significant release of TNF-α and IL-6 in sensitized lymphocytes. This suggests that the E variant may either harbor unique immunogenic epitopes or alter the proteolytic release of peptides during digestion. However, the possibility cannot be excluded that the observed effect results not from the action of a single protein variant alone, but rather from a unique repertoire of peptides released during digestion of specific combinations of protein variants in the already sensitized environment. While our findings underscore the pro-inflammatory potential associated with specific genotypes, further mechanistic studies are essential to determine whether the observed effects stem directly from the E variant or arise from complex interactions within the milk protein matrix and its digestion profile.

## Figures and Tables

**Table 1:** Genotypes of cows involved in the study.

Pooled Milk Symbol	Cows’ Genotype		
	*CSN2*	*CSN3*	*BLG*
REF	A1A2	AB	AB
HM1	A1A1	AA	AA
HM2	A2A2	BB	BB
HT1	A1A1	AE	AA, BB or AB ^1^
HT2	A1A1	BE	AA, BB or AB ^1^

*CSN2*—gene coding β-casein, *CSN3*—κ-casein, *BLG*—β-lactoglobulin. ^1^ Variants of the *BLG* gene in the group included homozygotes AA and BB, and the AB heterozygote, giving the AB β-lactoglobulin phenotype.

**Table 2:** Contribution of experimental factors—group (sensitization state of lymphocytes) and treatment (milk variants)—to the total variance (η^2^) of proliferation, lymphocyte phenotypes, and cytokine levels.

Variable	*P* Group	η^2^ Group	*P* Treatment	η^2^ Treatment	*P* Interaction	η^2^ Interaction
Proliferation	<0.0001	0.21	<0.0001	0.30	<0.0001	0.20
CD4^+^CD25^+^	<0.0001	0.69	0.1081	0.04	0.0019	0.10
CD4^+^Foxp3^+^	<0.0001	0.40	0.9075	0.01	<0.0001	0.34
IL-17A	0.0575	0.04	0.0155	0.23	0.0646	0.17
TNF-α	<0.0001	0.25	<0.0001	0.45	<0.0001	0.30
IL-6	<0.0001	0.31	<0.0001	0.34	<0.0001	0.35
IL-4	0.5761	0.00	0.0562	0.22	0.5818	0.08
IL-2	0.2833	0.02	0.5197	0.08	0.0112	0.27
IL-10	0.0557	0.05	0.0994	0.16	0.0613	0.19
IFN-γ	0.0403	0.06	0.2585	0.13	0.1441	0.16

## Data Availability

The original contributions presented in this study are included in the article and Supplementary Material. Further inquiries can be directed to the corresponding author.

## Supplementary Materials

The following supporting information can be downloaded at: https://www.mdpi.com/article/10.3390/nu18183021/s1, Figure S1: Schematic workflow for the generation of genotype-defined bovine milk digests; Figure S2: Schematic overview of the murine casein-sensitization model and the subsequent ex vivo splenocyte experiment; Figure S3: Simulated gastro-intestinal digestion (SDG) of (A) reference milk (REF) and enzymatic control of the digestion (ENZ), (B) homozygotic milks (HM1 and HM2) and (C) heterozygotic milks (HT1 and HT2). (D) Comparison of centrifuged digests (CDs) and enzyme preparations used for SDG (PEP—pepsin, PAN—pancreatin). BD—before digestion, GS—gastric stage, IS—intestinal stage, CD—centrifuged digests, PEP—pepsin enzyme, PAN—enzymes in pancreatin; Figure S4: Chromatograms of milk digests from reference milk (REF), homozygotic variants (HM1 and HM2), and heterozygotic variants (HT1 and HT2). For comparison, chromatograms of digested milk proteins—α-casein, κ-casein, β-lactoglobulin—as well as a casein mixture are also shown; Figure S5: Secretion of cytokines by casein-sensitized (CAS, white bars) and non-sensitized (PBS, grey bars) lymphocytes stimulated ex vivo with digests of the tested cow’s milk variants. Mean fluorescence intensity values recorded for (A) IL-4, (B) IL-2 and (C), IL-17A produced by lymphocytes following treatment with milk hydrolysates (REF, HM1, HM2, HT1, HT2), medium alone, enzymatic control (ENZ), or concanavalin A (ConA); Figure S6: Gene expression in CAS splenocytes stimulated ex vivo with digests of reference milk (REF), homozygotic variants (HM1, HM2), heterozygotic variants (HT1, HT2), and the enzymatic digestion control (ENZ). Values significantly different at *p* < 0.05 are indicated with small letters; Table S1: Characteristics of the milk protein gene variants analyzed in the study; Table S2: Primers applied in the study.

## Abbreviations

The following abbreviations are used in this manuscript:

CMPA	Cow milk protein allergy
*CSN1S1*	Casein alpha-S1 gene
*CSN2*	Beta-casein gene
*CSN3*	Kappa-casein gene
*BLG*	Beta-lactoglobulin gene
*LALBA*	Alpha-lactalbumin gene
