# Supplementary material for: Ex Vivo Immune Response of Casein-Sensitized Lymphocytes to Bovine Milk Protein Composite Genotypes: Beyond the β-Casein A1/A2 Paradigm
Source: Nutrients. 2026 Sep 16;18(18):3021. doi: 10.3390/nu18183021 (PMC13610451; doi:10.3390/nu18183021)
Supplement: Supplementary file 1 [file nutrients-18-03021-s001.zip › nutrients-4490223-supplementary.pdf]

Figure S1. Schematic workflow for the generation of genotype-defined bovine milk digests.

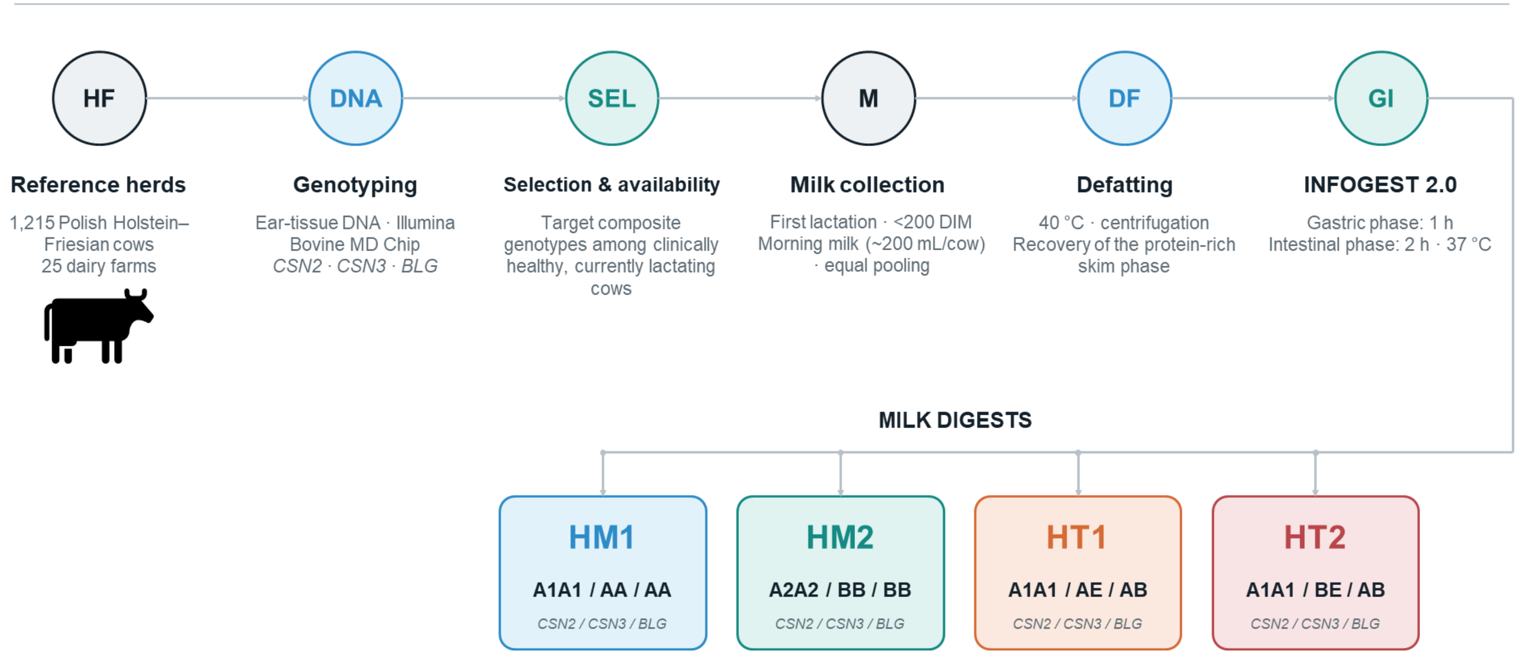

A reference population of 1,215 Polish Holstein–Friesian cows from 25 dairy farms was genotyped using ear-tissue DNA and the Illumina Bovine MD Chip. Composite genotypes of genes encoding β-casein (*CSN2*), κ-casein (*CSN3*), and β-lactoglobulin (*BLG*) were selected according to genotype frequency and the availability of clinically healthy lactating cows. Milk collected from cows representing the selected genotypes was pooled in equal proportions, defatted by centrifugation, and subjected to simulated gastrointestinal digestion according to the INFOGEST 2.0 protocol. Following heat inactivation of enzymes and clarification by centrifugation, soluble milk digests representing HM1 (A1A1/AA/AA), HM2 (A2A2/BB/BB), HT1 (A1A1/AE/AB), and HT2 (A1A1/BE/AB) composite genotypes were obtained. Genotypes are presented in the order *CSN2/CSN3/BLG*.

Figure S2. Schematic overview of the murine casein-sensitization model and the subsequent *ex vivo* splenocyte experiment.

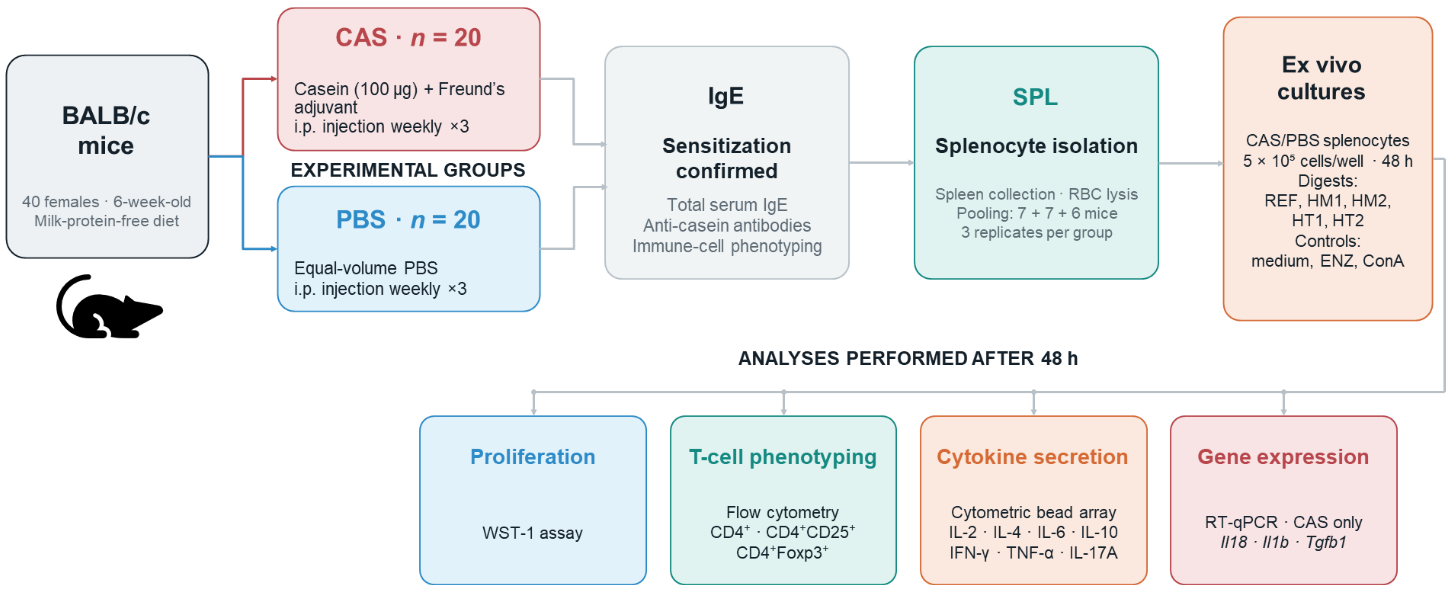

Female BALB/c mice were assigned to a casein-sensitized group (CAS, $n = 20$), receiving three weekly intraperitoneal injections of casein with Freund's adjuvant, or to a non-sensitized control group (PBS, $n = 20$), receiving an equivalent volume of phosphate-buffered saline. Sensitization was verified based on serum IgE, anti-casein antibody levels, and immune-cell phenotyping. Splenocytes were isolated and pooled from 7, 7, and 6 mice, providing three biological replicates per group. CAS and PBS splenocytes were cultured for 48 h with digests of reference milk (REF) and genotype-defined milks (HM1, HM2, HT1, and HT2). Medium alone, the enzymatic digestion control (ENZ), and concanavalin A (ConA) were included as controls. Following incubation, lymphocyte proliferation was assessed using the WST-1 assay, T-cell populations were characterized by flow cytometry, and cytokine secretion was determined using a cytometric bead array. The expression of *Il18*, *Il1b*, and *Tgfb1* was analyzed by RT-qPCR in CAS splenocytes only.

Figure S3. Simulated gastro-intestinal digestion (SDG) of (A) reference milk (REF) and enzymatic control of the digestion (ENZ), (B) homozygotic milks (HM1 and HM2) and (C) heterozygotic milks (HT1 and HT2). (D) Comparison of centrifuged digests (CDs) and enzyme preparations used for SDG (PEP—pepsin, PAN—pancreatin). BD—before digestion, GS—gastric stage, IS—intestinal stage, CD—centrifuged digests, PEP—pepsin enzyme, PAN—enzymes in pancreatin.

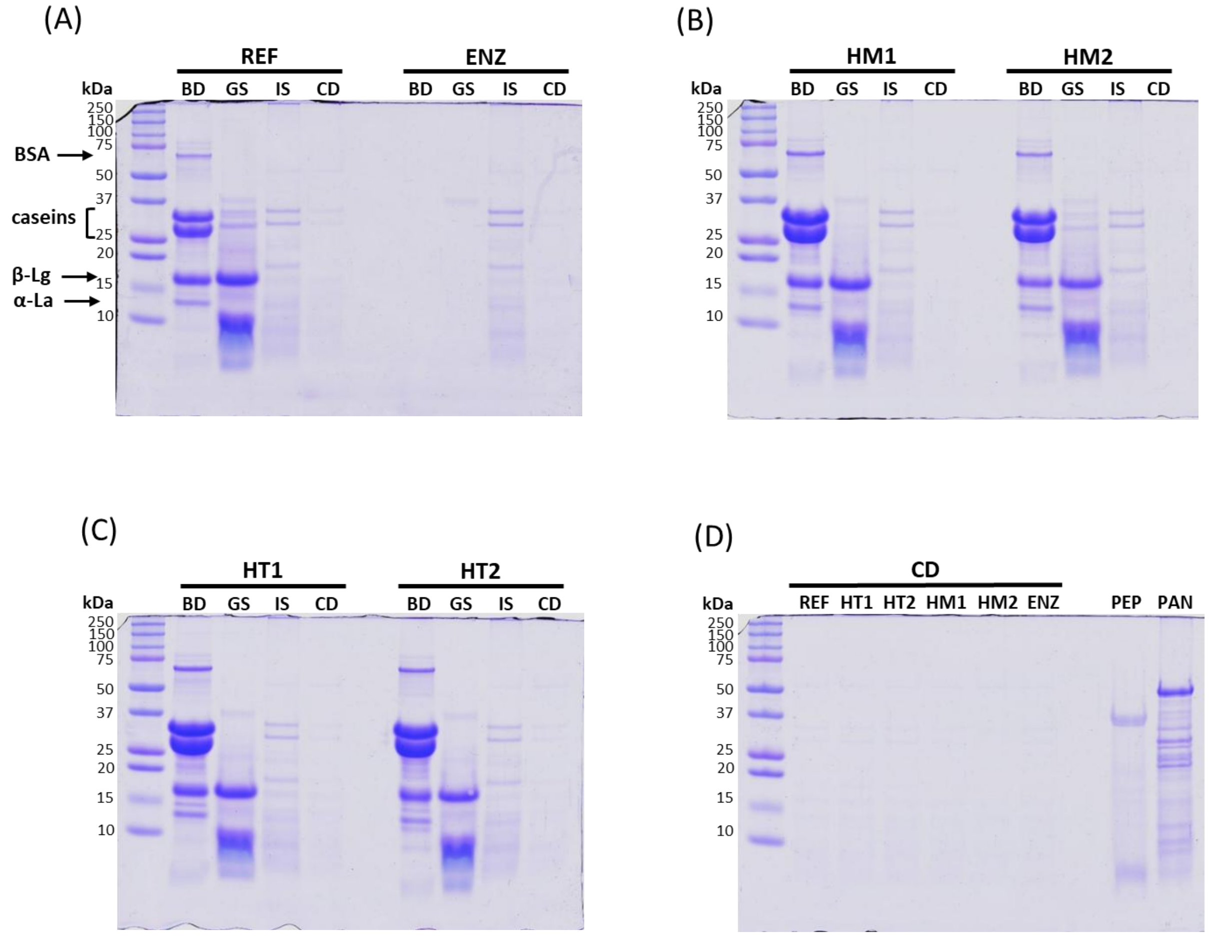

Figure S4. Chromatograms of milk digests from reference milk (REF), homozygotic variants (HM1 and HM2), and heterozygotic variants (HT1 and HT2). For comparison, chromatograms of digested milk proteins—α-casein, κ-casein, β-lactoglobulin—as well as a casein mixture are also shown.

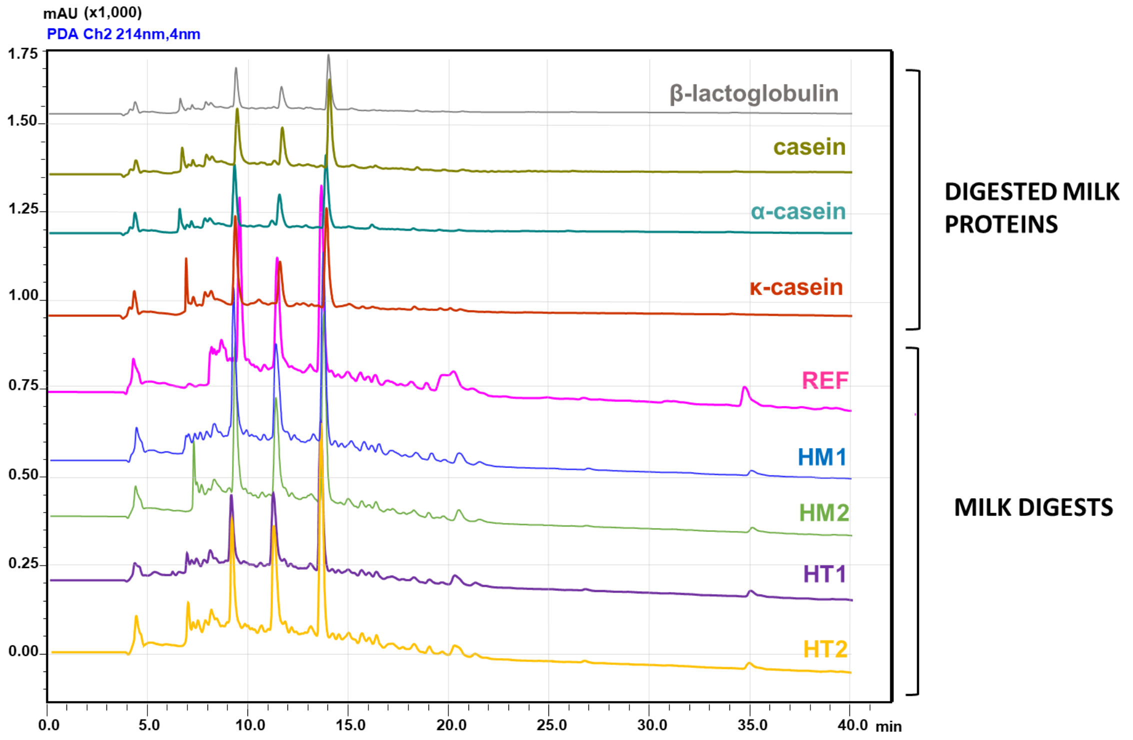

Figure S5. Secretion of cytokines by casein-sensitized (CAS, white bars) and non-sensitized (PBS, grey bars) lymphocytes stimulated ex vivo with digests of the tested cow's milk variants. Mean fluorescence intensity values recorded for (A) IL-4, (B) IL-2 and (C), IL-17A produced by lymphocytes following treatment with milk hydrolysates (REF, HM1, HM2, HT1, HT2), medium alone, enzymatic control (ENZ), or concanavalin A (ConA).

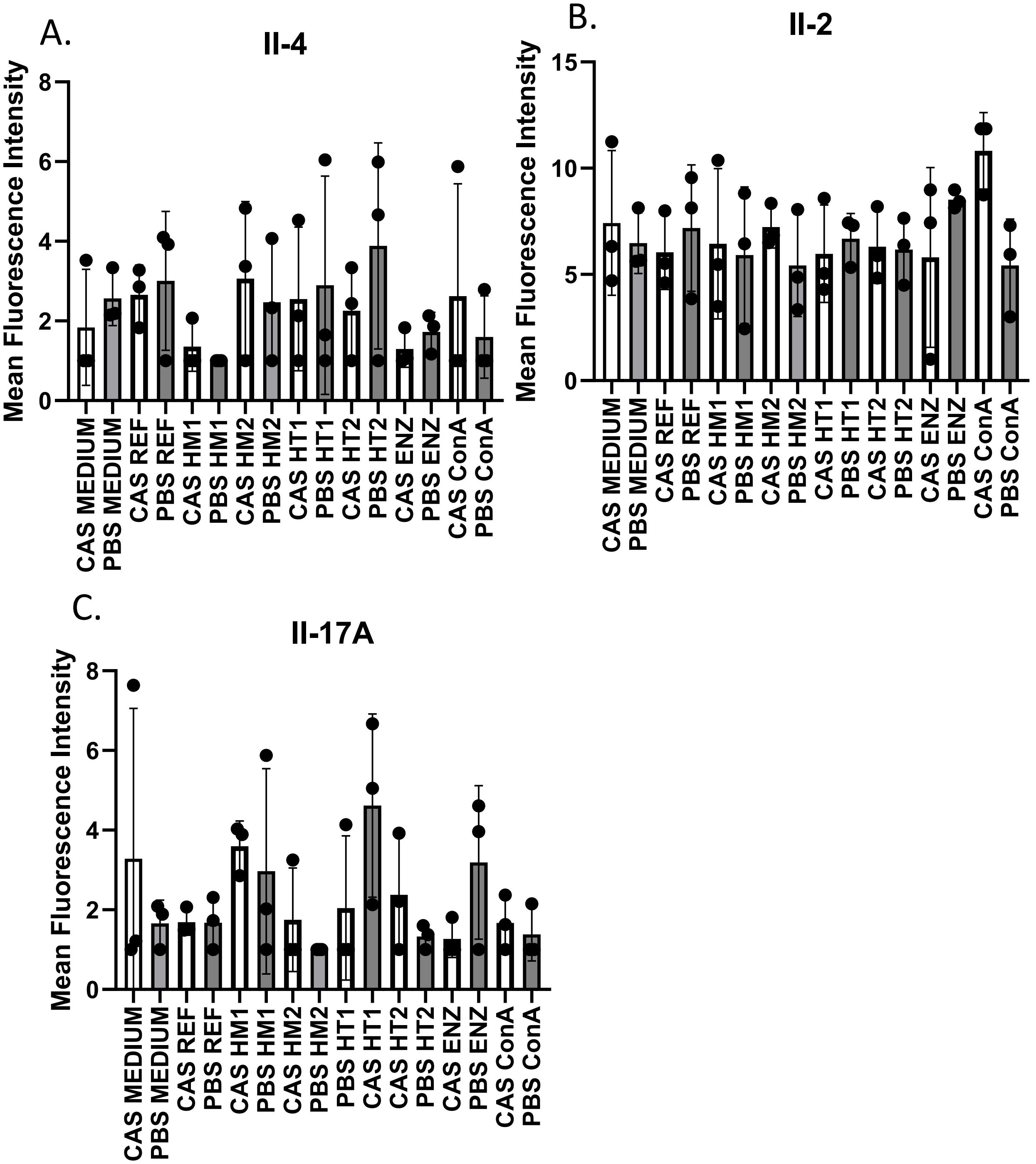

Figure S6. Gene expression in CAS splenocytes stimulated ex vivo with digests of reference milk (REF), homozygotic variants (HM1, HM2), heterozygotic variants (HT1, HT2), and the enzymatic digestion control (ENZ). Values significantly different at $p < 0.05$ are indicated with small letters.

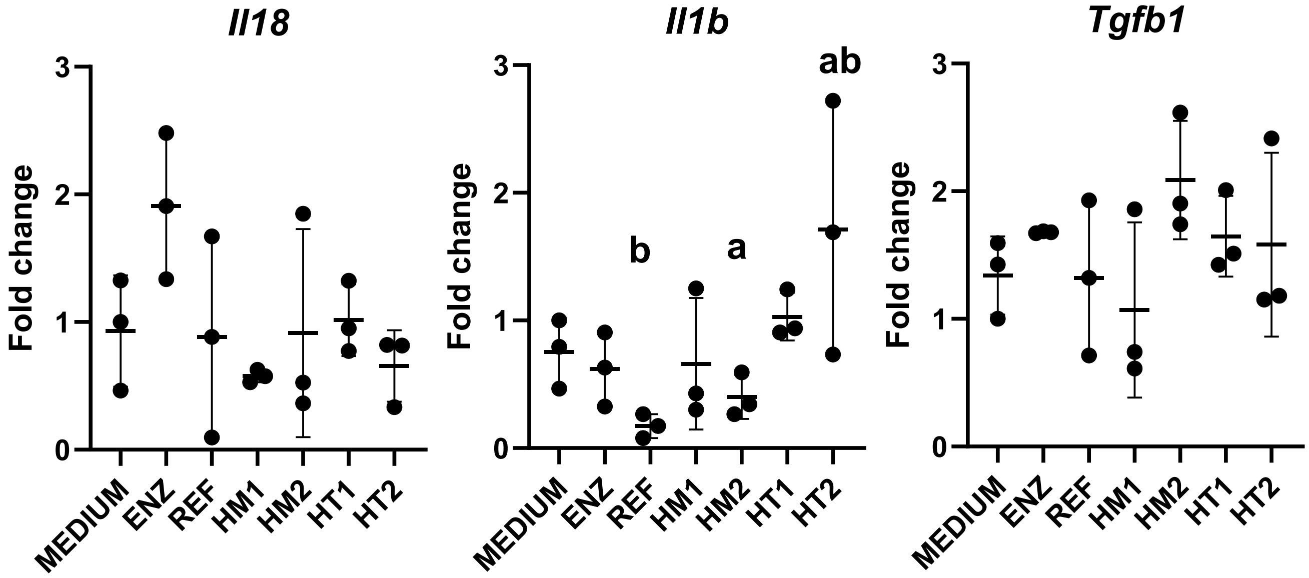

Table S1. Characteristics of the milk protein gene variants analyzed in the study.

**Gene**	**Variant**	**Amino acid substitution**	**Variant ID**
CSN2	A2/A1	His67Pro	rs43703011
CSN3	A/B	Thre136Ile	rs43703015
CSN3	A/B	Asp148Ala	rs43703016
CSN3	E	Ser155Gly	rs43703017
BLG	A/B	Asp64Gly	rs110066229
BLG	A/B	Val118Ala	rs109625649

Table S2. Primers applied in the study.

Name	Sequence	Target
mIL-18f	actgtacaaccgcagtaatacgg	Mouse interleukin 18
mIL-18r	gagtgaacattacagatttatccc	Mouse interleukin 18
mIL-1BRf	ttgacggaccccaaaagatg	Mouse interleukin 1
mIL-1BRr	agaaggtgctcatgtcctcat	Mouse interleukin 1
mTGF-B1f	agacggaatacagggtttcgattca	Mouse transforming growth factor beta 1
mTGF-B1r	cttgggcttgcgacccacgtagta	Mouse transforming growth factor beta 1
